# Impact of Microscopic Confirmation on Therapeutic Management of Pancreatic Cancer Patients: Lessons from an Italian Regional Tumor Registry

**DOI:** 10.3390/cancers14215372

**Published:** 2022-10-31

**Authors:** Alberto Fantin, Mario Gruppo, Ottavia De Simoni, Sara Lonardi, Chiara Cristofori, Tiziana Morbin, Giulia Peserico, Sabina Grillo, Annalisa Masier, Monica Franco, Pierluigi Pilati, Stefano Guzzinati, Manuel Zorzi, Massimo Rugge

**Affiliations:** 1 Department of Gastroenterology, Veneto Institute of Oncology IOV-IRCCS, 35128 Padua, Italy; 2 Unit of Surgical Oncology of Digestive Tract, Veneto Institute of Oncology IOV-IRCCS, 35128 Padua, Italy; 3 Medical Oncology Unit 3, Department of Oncology, Veneto Institute of Oncology IOV-IRCCS, 35128 Padua, Italy; 4 Veneto Tumor Registry, Azienda Zero, 35128 Padua, Italy; 5 Pathology and Cytopathology Unit, Department of Medicine-DIMED, University of Padua, 35128 Padua, Italy

**Keywords:** pancreatic cancer, incidence, microscopic diagnosis, survival, epidemiology

## Abstract

**Simple Summary:**

In consideration of poor survival and rising incidence of pancreatic cancer (PC), the impact of microscopic diagnosis of PC (MiDPC) turns out to be crucial in therapeutic choice. Clinical and oncological data regarding all cases of PC in the Veneto region (Italy) from 1987 were extracted from the Veneto cancer registry. The percentage of MiDPC was 60% in 2018. MiDPC was higher among patients <75 years old (84.4%) compared to those ≥75 years old (38.9%). Patients with MiDPC had higher 5-year survival than patients with no MiDPC (12.9% vs. 1.2%, *p* < 0.001). Considering the evidence regarding the impact of histopathological parameters on PC patient selection and tailored treatment approaches, 41% of PCs in the Veneto region are still not histologically proven, with a consequent impact on the choice of the correct treatment strategy and, thus, survival. In light of the increasing incidence of the disease, its mortality, and its heterogeneity, all efforts should be aimed to optimize diagnostic–therapeutic pathways, encouraging MiDPC.

**Abstract:**

Background: Incidence of pancreatic cancer (PC) is increasing worldwide and is set to become the second leading cause of cancer-related death in 2040 with a poor 5-year overall survival (OS). The aim of this study was to analyze the impact of microscopic diagnosis of PC (MiDPC) on diagnostic–therapeutic management and outcome. Methods: The Veneto region (north-eastern Italy) has been covered by a cancer registry (CR) since 1987. Clinical and oncological data about all cases of PC in the Veneto region from 1987 were extracted from the Veneto CR database. Results: In 2018, 1340 incident cases of PC in the Veneto population were registered (4.1% of all malignant tumors), with an increasing trend in females and stable incidence in males. Five-year OS in patients with PC was 8%. The percentage of MiDPC increased from 44% in 2010 to 60% in 2018 (*p* = 0.001). MiDPC was higher among patients aged < 75 years old (84.4%) compared to those aged ≥75 years old (38.9%), *p* = 0.001. Between 2010 and 2018, a significant increase in biopsy on the primary neoplasm (24.9% vs. 13%, *p* < 0.001) was reported. Patients with MiDPC had higher 5-year survival than patients with no MiDPC (12.9% vs. 1.2%, *p* < 0.001). Conclusions: The implementation of MiDPC was essential to improve diagnostic–therapeutic pathways and consequently the survival of PC patients.

## 1. Introduction

Pancreatic cancer (PC) is one of leading causes of cancer mortality in developed countries despite, being the 10th most common form of cancer [1]. Due to the advanced stage at diagnosis, about 80% of patients have an unresectable tumor, and long-term survival after surgical resection is poor [2,3,4]. In the last decade, overall survival (OS) in PC patients has slightly improved because of the wider use of systemic chemotherapy, the use of combination chemotherapies, and the improvement of surgical techniques [5,6,7]. Nevertheless, 5-year OS in PC patients is 8% (ranging from 2% to 9%) [7,8,9].

Globally, a total of 458,918 new cases of PC and 432,242 related deaths were reported in 2018, with a global age standardized incidence rate of 4.8 per 100,000 [10]. Moreover, Huang et al. reported an overall increasing trend in PC incidence and mortality in the past decade, especially among women and the older population [11].

Given the rise in incidence and mortality trends, the improvement of early diagnosis and selection of the correct treatment strategies of PC should be assigned as a top priority in policy agendas and clinical guidelines in order to reduce mortality. In fact, recent evidence illustrates the importance of understanding the impact of the complex microenvironment components in tumor suppression and progression, as well as of the mutational status of PC in order to achieve personalized therapeutic pathways and to accelerate progress towards more effective treatment strategies [12,13]. 

Considering the rising disease burden and this last recent evidence, Endoscopic UltraSound (EUS), as well as improvement in diagnostic tools and percutaneous techniques, play an important role in early detection of PC in optimizing the staging of PC, in terms of the best histological definition of premalignant and malignant pancreatic lesions and the best characterization of molecular parameters of PC [14,15,16,17,18]. Consequently, the impact of microscopic diagnosis of PC (MiDPC), defined as the histopathological diagnosis obtained by both primary lesions and metastases, turns out to be crucial in staging the PC patients for the purpose of optimizing therapeutic choice.

The aim of this study was to analyze the incidence of PC and the impact of MiDPC on diagnostic-therapeutic management and outcomes in the Veneto region of Italy.

## 2. Materials and Methods

The Veneto region (north-eastern Italy; population of 4.9 million inhabitants in 2018) has been covered by a cancer registry (CR) since 1987 [19,20]. The Veneto CR initially covered about one-third of the regional population; over time, registration coverage progressively increased reaching about a half of the population in 2010 and 100% since 2014. The Veneto CR collects the following information for each patient with a diagnosis of cancer: date of birth, date of diagnosis, gender, vital status, site of primary tumor, morphology code, and diagnostic confirmation. All cases of pancreatic cancer (International Classification of Diseases, 10th revision: C25) diagnosed in 2010 and 2018 were extracted from the Veneto CR database and considered in this study. All hospital discharges from 1 January 2010 to 31 December 2011 of patients with a pancreatic cancer diagnosed in 2010 and from 1 January 2018 to 31 December 2019 for those diagnosed in 2018, with pancreatic resection intervention codes (according to International Classification of Diseases, Ninth Revision, Clinical Modification (ICD-9-CM) diagnostic codes 52.2, 52.21, 52.22, 52.51, 52.52, 52.53, 52.59, 52.6, 52.7), were extracted from the regional archive of hospital discharge records. Both hospital discharges and regional outpatient services databases for the same periods were accessed to collect information about chemotherapy (ICD-9-CM procedure code 99.25 or V58.11 diagnosis code). All patients with histologically confirmed diagnosis of PC on the primary tumor or on the metastases were considered in the MiDPC group. Microscopic confirmation of pancreatic cancer was obtained by endoscopic, percutaneous, or surgical specimens.

### 2.1. Statistical Analysis

Descriptive statistics were used to summarize the main characteristics of the two study cohorts (cases incident in 2010 and 2018). The chi-squared test was used to determine the association between categorical variables. For binary variables, we also calculated the relative risk with 95% confidence interval. We used the long-rank test to compare differences in survival between groups. Statistical significance was set at 0.05. All the analyses were performed using SAS Enterprise Guide, V.6.1 statistical package (SAS Institute, Cary, NC, USA).

### 2.2. Ethics

The Italian legislation identifies cancer registries as collectors of personal data for surveillance purposes without explicit individual consent. We did not require approval from a research ethics committee as this study was a descriptive analysis of individual data without any direct or indirect intervention on patients [21].

## 3. Results

### 3.1. Incidence

The incidence rate of malignant tumors (all sites but non-melanomatous skin cancers) in the Veneto population in 2018 was 32,313 cases (16,975 in men and 15,338 in women). The top three cancer sites in men were prostate (3855 cases, 22.7%), lung (1962, 11.6%), and colon–rectum (1867, 11%), and in women were breast (4924 cases, 32%), colon–rectum (1642 cases, 10.7%), and lung (1064 cases, 6.9%). In 2018, the number of incident cases of PC was 1340 (4.1% of all malignant tumors), as reported in Table 1. Incidence rates of PC were 28.6 × 100,000 in men and 26.3 × 100,000 in women. PC was the seventh most common cancer overall in men (627 cases, 3.7%) and the fifth most in women (713 cases, 4.6%). The average age at PC diagnosis was 74 (standard deviation of 11.5) years. A total of 55% of PC patients were ≥75 years. Table 1 compares the main data about PC patients in 2018 with those in 2010. 

**Figure 1:**
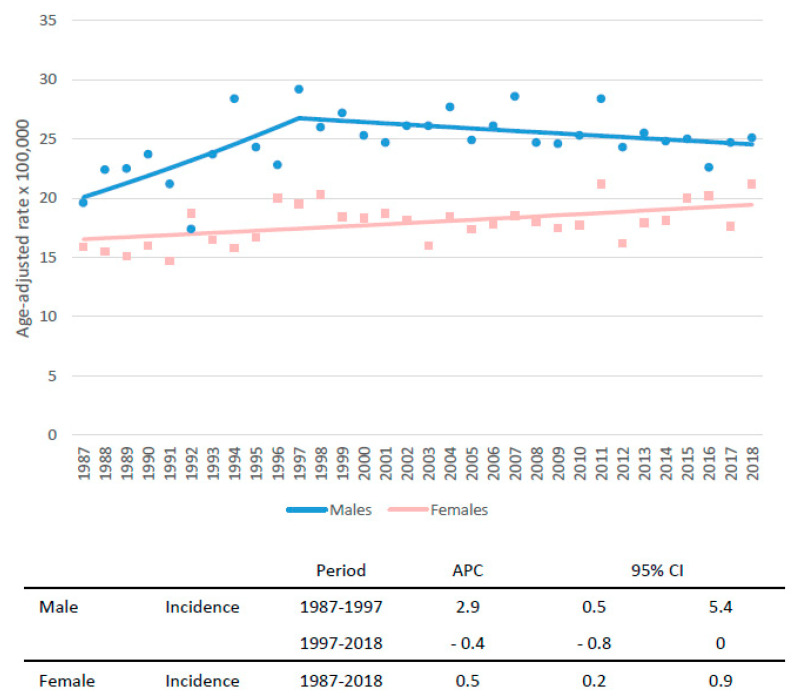
Trend of incidence rates of pancreatic cancer incidence with annual percent change (APC) and 95% confidence interval (CI) in the Veneto region population by sex.

Figure 1 shows the trend of PC incidence rates by sex from 1987 to 2018 in the Veneto population. Compared to the early 1990s, the incidence rates increased both in men (from 21.5 × 100,000 in 1987–1989 to 24.1 × 100,000 in 2016–2018) and in women (from 15.5 to 19.7). A steep increase was recorded in men until 1997 (annual percent change (APC): +2.9), followed by a not significant decrease (APC − 0.4). In women, a slight, although statistically significant, increase in PC incidence was reported over the 30 years of observation (APC + 0.5).

**Figure 2:**
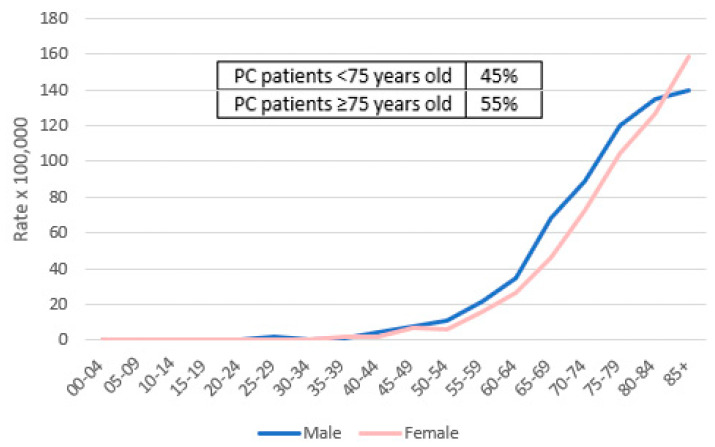
Incidence rate of pancreatic cancer in the Veneto region population by age and sex in 2018.

Figure 2 shows the incidence rate of PC by sex and age in 2018.

### 3.2. Diagnosis

**Figure 3:**
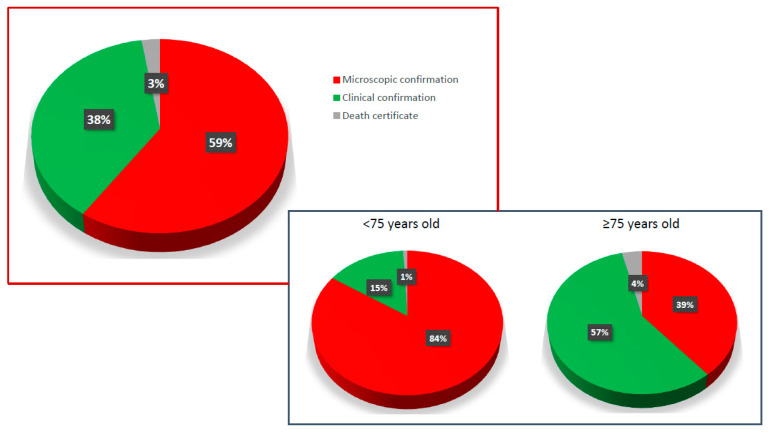
Types of diagnostic confirmation for PC patients of the Veneto region population. Microscopic confirmation included all PC patients with histological confirmation, obtained either by biopsy on primary lesions or metastases, or by surgery. Clinical confirmation included all cases of PC patients without histological diagnosis.

The proportion of cases that were microscopically confirmed increased from 37.6% in 1990–1992 to 44.7% in 2000–2002 and to 59.5% in 2018. As shown in Figure 3, in 2018, microscopic confirmation was reported in 59% of PC cases, clinical confirmation in 38%, and confirmation only by death certificate in 3%. The MiDPC was significantly higher among patients <75 years old (84.4%) compared to those ≥75 (38.9%) (*p* = 0.001). On the contrary, clinical confirmation of PC cases was higher among patients older than 75 years old (57%) compared to those <75 years old (15%).

**Figure 4:**
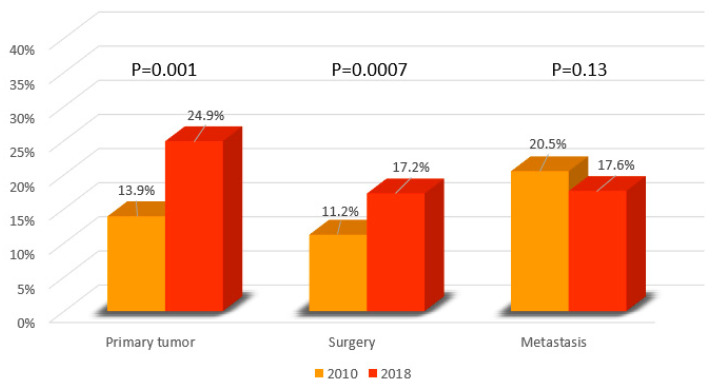
Type of diagnostic microscopic confirmation in 2010 and 2018 in PC patients in the Veneto region population (percentage expressed on total PC cases of the referral year).

Microscopic confirmation of PC cases could be indifferently obtained with a biopsy of the primary tumor, a biopsy of metastasis, or with surgery. In 2010, MiDPC was based on biopsy of metastasis in 20.5%, on biopsy of the primary neoplasm in 13.9%, and on resective surgery in 11.2% (Figure 4).

In 2018, a significant increase in biopsy on primary neoplasm and in resective surgery was reported, compared to 2010 (24.9% vs. 13.9%, *p* < 0.001 and 17.2% vs. 11.2%, *p* < 0.001, respectively). A slight decrease in diagnostic confirmation on metastasis was observed between 2010 and 2018 (20.5% vs. 17.6%, respectively). In 2018, MiDPC by biopsy on primary neoplasm was significantly higher among patients younger than 75 years old compared to those ≥75 years old (34.7% vs. 16.8%, *p* < 0.001). In 2018, patients younger than 75 years old underwent diagnosis on primitive neoplasm 2.5 times more than patients ≥75 (34.7 vs. 16.8%, *p* < 0.001), as reported in Table 2. All data about MiDPC are reported in Table 2. 

**Figure 5:**
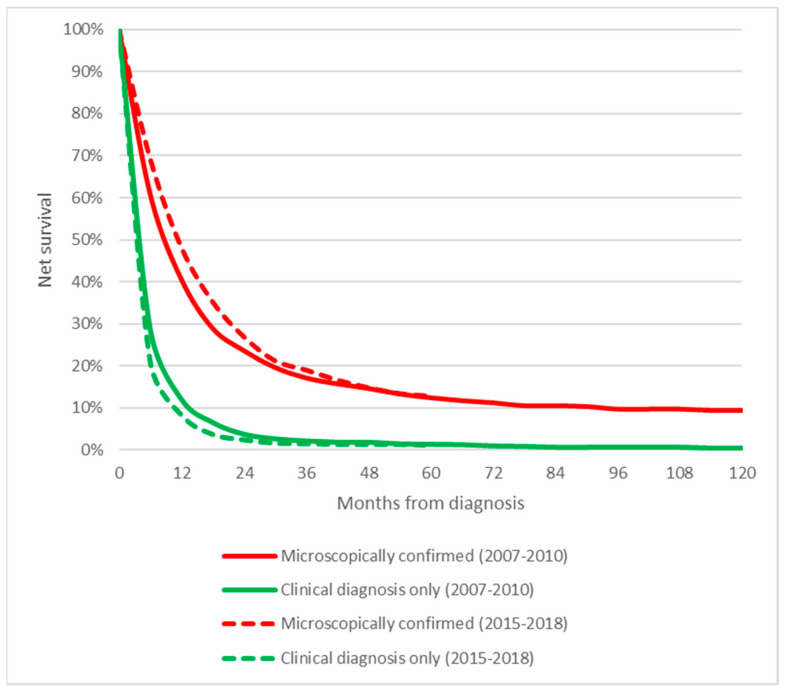
Survival of pancreatic cancer patients of the Veneto region population according to diagnostic confirmation and period.

As shown in Figure 5, PC patients with MiDPC had a higher survival rate than patients with clinical confirmation. Five-year survival of patients with MiDPC was 12.9%, significantly higher than patients with clinical confirmation (1.2%) (*p* < 0.001). 

**Figure 6:**
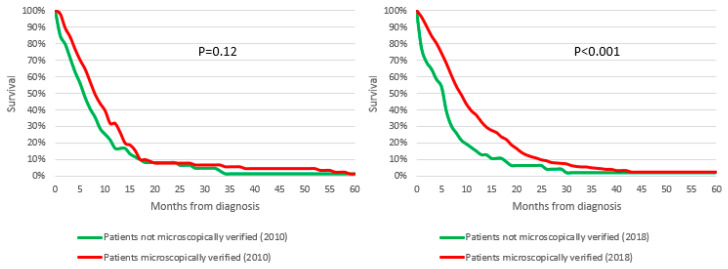
Survival of Stage III-IV pancreatic cancer patients of the Veneto region population who underwent chemotherapy alone according to diagnostic confirmation and year.

As shown in Figure 6, after stratification for stage and therapeutic treatment, Stage III-IV PC patients with MiDPC who underwent chemotherapy alone (without surgical treatment) had a higher survival than patients who underwent the same treatment without MiDPC. This difference was particularly marked in 2018 with 1 year OS of 36.9% in the MiDPC group versus 15% in patients without MiDPC (*p* = 0.003) and 2-year OS of 11.1% vs. 6%, respectively (*p* < 0.001).

**Figure 7:**
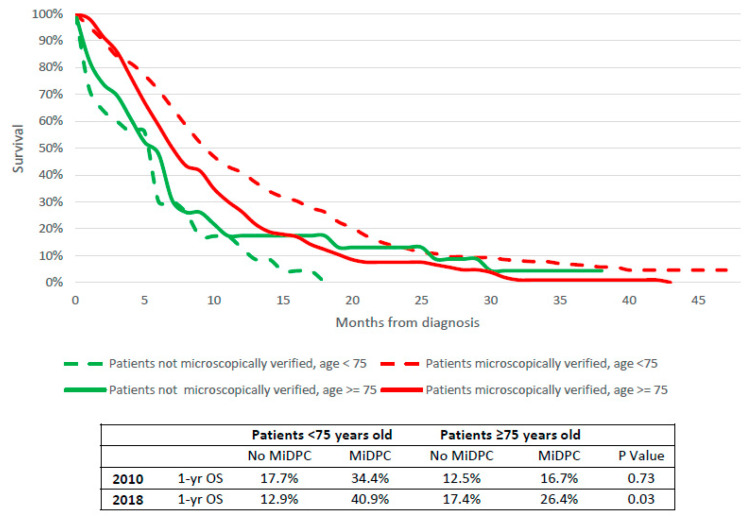
Survival of Stage III-IV pancreatic cancer patients of the Veneto region population who underwent chemotherapy alone according to diagnostic confirmation and age.

As reported in Figure 7, these data were also confirmed after stratification for age; both Stage III-IV PC patients <75 years old and older subjects with MiDPC who underwent chemotherapy had a 1-year OS higher than patients without MiDPC. This difference was particularly marked in 2018 (40.9% vs. 12.9%; 26.4% vs. 17.4% respectively, *p* = 0.03). 

## 4. Discussion

GLOBOCAN estimated 458,918 (2.5%) cases of PC worldwide in 2018 [22]. In the same year, in the Veneto region, 1340 new cases of PC were reported (4% of all cancer sites); PC was the seventh most common cancer. Incidence rates in 2018 was 28.6 per 100,000 among males and 26.3 per 100,000 among females. As reported by estimated projection of US cancer incidence and death by Rahib et al., the incidence of PC is expected to increase [23]. During the next 20 years, PC is expected to increase from third to second causes of death among cancers, with a projection of 46,000 deaths in year [23]. The substantial increase in the incidence and mortality of PC may indicate a growing prevalence of its risk factors associated with globalization, urbanization, and economic development [11].

The trend of PC incidence rates by sex from 1987 to 2018 in our study confirmed a gradual increase, particularly among females. Similar figures were reported in the US, where Gaddam et al. observed in the last decades a significantly greater relative increase in incidence of PC cases among women younger than 55 years (APC 1.93% (95% CI, 1.57–2.28%)) compared with men younger than 55 years (0.77% (95% CI, 0.50–1.05%)) with non-equal trends (*p* = 0.002) [24]. A recent study identified the main risk factors for early onset PC in genetic mutations, smoking, obesity, and metabolic disease [25]. Obesity and metabolic syndrome have been reported as major factors that could lead to a significant increase in PC incidence, particularly in the female population [26]. 

Because of the rising disease burden, there is an increasing emphasis on early identification of PC and premalignant pancreatic lesions in high-risk individuals. As shown by Blackford et al, factors such as earlier diagnosis and enrollment into pancreatic surveillance programs have already contributed in the US to the recent increase in diagnoses of stage IA PC [27]. In this regard, technical and technological improvement both for non-invasive imaging (CT scan, MRI, nuclear imaging) and minimally invasive imaging (EUS, endoscopic retrograde cholangiopancreatography) represent crucial tools in early detection and staging of PC. 

In our study, 59.5% of patients had MiDPC in 2018. This percentage increased from 37.6% in 1990-1992, attempting to keep pace with improved staging and therapeutic strategies in PC. Understandably, the MiDPC were higher among patients younger than 75 years (84%) compared to those ≥75 (39%). 

Between 2010 and 2018, a significant increase in MiDPC by biopsy on primary neoplasm and by surgery (24.9% vs. 13.9%, *p* < 0.001 and 17,24% vs. 11.9%, *p* = 0.0007, respectively) was reported. These data go hand in hand with the improvement of percutaneous techniques, such as ultrasound- and CT-guided biopsies, as well as an increase in resective and diagnostic surgery, in particular after the introduction of neoadjuvant chemotherapy protocols [28,29,30]. The increase in the use of EUS has been conditioned not only by the improvement of technology and the advent of neoadjuvant therapy but is also the consequence of the change in guidelines from the ASGE that recommends EUS rather than ERCP for evaluation of PC, reflecting the evidence of an improved sensitivity and specificity of EUS as well as its capacity to obtain histological specimens [31]. As reported by Rustgl et al., the use of EUS in PC in US increased from 11.6% in 2003 to 31.4% in 2016 in PC patients [32]. As expected, patients with loco-regional disease or patients who received chemotherapy or radiation were more likely to receive EUS [32]. Moreover, social, economic, and demographic factors conditioned the use of EUS [32]. 

The benefits of the use of EUS in diagnosis and staging of PC are nowadays well established. As reported by Wang et al., EUS-FNA may not only provide sufficient tissue to allow pre-treatment histological diagnosis, but also deeper analysis on the mutational status and immunological pancreatic microenvironment [33]. In fact, as reported by Ngamruengphong et al., EUS evaluation was independently associated with improved survival, and with increased curative-intent surgery and chemoradiation treatment [34]. Rustgl et al. confirmed that EUS resulted in protection against mortality [32]. 

The survival advantage of undergoing EUS should be researched in identifying patients with advanced PC who would not benefit from resection and therefore avoid the morbidity and mortality related to unnecessary surgery, as reported by Welch et al., with the detection of potentially resectable disease by rendering more patients eligible for curative surgery [35]. Moreover, the accuracy of EUS in studying a possible vascular invasion has been reported up to 82%, compared with CT’s accuracy of 79% [15]. Furthermore, EUS with fine-needle aspiration (EUS-FNA) plays an essential role not only in diagnosing PC but also in diagnosing the correct etiology for solid pancreatic masses, with sensitivity of over 85% and specificity of 96%, in order to avoid unnecessary pancreatic surgery and its known high morbidity rates [16,17,36,37]. Finally, accumulating evidence illustrates the importance of achieving a tailored therapeutic approach that is based upon the comprehension of the multi-faceted roles of the complex tumor microenvironment components and mutational status in PC, which currently only MiDPC can offer [12].

The introduction of poly chemotherapy regimens has been associated with significant improvement in oncological outcomes, both in localized and advanced disease [6]. Furthermore, BRCA mutation has been related to tumor-platin-sensitivity and has recently been correlated with a specific therapeutic option, such as the use of a specific PARP inhibitor, with significant progression-free-survival in metastatic disease [38].

On the contrary, the SMAD4 mutation is reported to be associated with poor prognosis [39]. This evidence suggests that a deep knowledge of tumor biology is mandatory to reach better outcomes. Histopathological and molecular analyses of neoplastic tissue are and will become increasingly crucial for the choice of the best treatment option available. In clinical practice, CA 19-9 is already commonly adopted as a marker of PC aggressiveness in the choice of therapeutic treatment, besides common radiological parameters of resectability [40]. Furthermore, molecular subtypes of PC have been demonstrated to be related to different tumorigenesis and evolutive pathways, with different chemosensitivity; potential therapeutic targets; and, finally, survival [41]. As reported by Dreyer et al., molecular subtypes should be considered in order to lead patient selection for surgery, as well as pathological and molecular parameters that could be useful for the choice of the best treatment option with improvement in oncological outcome [42]. Our data seem to support this evidence: even though surgical resection remains the best therapeutic option in localized disease (above all in combination with adjuvant or neoadjuvant chemotherapy), medical treatment in non-resectable disease seems to be significantly associated with histopathological analysis of neoplastic tissue, with a gain of survival compared to not histologically proven cancers, particularly in the first year of treatment (Figure 6). These findings are also confirmed after stratification for age, suggesting a survival benefit for MiDPC both in younger and elderly patients (Figure 7). Furthermore, as reported in Figure 6 and Figure 7, this evidence is more marked in 2018 than 2010, consequent to the introduction of EUS, polychemotherapy regimens and adoption of molecular markers in clinical practice in the Veneto region (since 2011 and 2012 respectively).

However, this study has some limitations to consider. Firstly, no specific clinical or oncological detailed data about patients were available; in particular, no specific data about criteria for the choice of chemotherapeutic regimens were retrievable from the regional registry; these data could have been important to further analyze the specific weight of MiDPC and should be investigated in specific trials in order to confirm or not confirm the hypothetic impact of MiDPC on oncological outcomes. In addition, survival analyses are probably affected by the younger age of the main part of the population with microscopic confirmation, which has an expected better survival regardless, due to a higher performance status than the clinical confirmation group.

In any case, considering the increasingly more marked evidence about impact of molecular and histopathologic factors on PC patient selection and tailored treatment approaches, 41% of patients with diagnosis of PC in the Veneto region still have no microscopic confirmation. These data are particularly evident in the population ≥75 years old, with 61% of subjects without MiDPC. As underlined by many authors, age should not be the determining factor in decisions regarding the best diagnostic and therapeutic approach: both the administration of adjuvant chemotherapy and surgical resection can be performed safely in elderly people with a proper pre-treatment assessment [43,44,45]. Certainly, these data could affect not only diagnostic efficacy but also the choice of the correct treatment strategy and, thus, survival. 

## 5. Conclusions

In light of the increasing incidence of the disease, its mortality, and heterogeneity, as well as the advancement on tumor biology knowledge and application in clinical practice, all efforts should be aimed to optimize and to tailor diagnostic–therapeutic pathways, encouraging the microscopic diagnosis of PC. 

## Figures and Tables

**Table 1:** Pancreatic cancers (PC) recorded in the Veneto region populations in 2010 and in 2018.

	Year of Diagnosis	
	2010	2018
Population covered by the Veneto cancer registry	2,581,953	4,880,936
No. of PC cases	590	1340
Crude rate of PC × 100,000	22.9	27.5
% of males	49%	47%
Average age (SD)	74 (11.6)	74 (11.5)
% of patients with <75 years	46%	45%
% of patients with ≥75 years	54%	55%

**Table 2:** Type of microscopic diagnosis in PC patients diagnosed in 2010 and in 2018 by age class (percentage expressed on total PC cases of the referral year).

	2010	2018	*p* Value
Biopsy of the primary tumor	13.9%	24.9%	<0.001
<75 years old	20.7%	34.7%	<0.001
≥75 years old	8.15%	16.8%	0.002
Surgery	11.19%	17.24%	0.007
<75 years old	19.9%	24.7%	0.14
≥75 years old	3.7%	10.9%	0.002
Biopsy of metastasis	20.5%	17.6%	0.13
<75 years old	26.9%	25.12%	0.5
≥75 years old	15%	11%	0.09

## Data Availability

The data presented in this study are available on request from the corresponding author.
